# DiNAMO: highly sensitive DNA motif discovery in high-throughput sequencing data

**DOI:** 10.1186/s12859-018-2215-1

**Published:** 2018-06-11

**Authors:** Chadi Saad, Laurent Noé, Hugues Richard, Julie Leclerc, Marie-Pierre Buisine, Hélène Touzet, Martin Figeac

**Affiliations:** 1 Univ. Lille, CNRS, Inria, UMR 9189 - CRIStAL - Centre de Recherche en Informatique Signal et Automatique de Lille, Lille, France; 2 Univ. Lille, Inserm, Lille University Hospital, UMR-S 1172 - JPARC - Centre de Recherche Jean-Pierre AUBERT, Lille, F-59000 France; 3 Sorbonne Université, UMR7238, Laboratory Computational and Quantitative Biology, LCQB, Paris, F-75005 France; 4 Univ. Lille. Plateau de génomique fonctionnelle et structurale, Lille, F-59000 France

**Keywords:** Motif, DNA, Chip-Seq

## Abstract

**Background:**

Discovering over-represented approximate motifs in DNA sequences is an essential part of bioinformatics. This topic has been studied extensively because of the increasing number of potential applications. However, it remains a difficult challenge, especially with the huge quantity of data generated by high throughput sequencing technologies. To overcome this problem, existing tools use greedy algorithms and probabilistic approaches to find motifs in reasonable time. Nevertheless these approaches lack sensitivity and have difficulties coping with rare and subtle motifs.

**Results:**

We developed DiNAMO (for DNA MOtif), a new software based on an exhaustive and efficient algorithm for IUPAC motif discovery. We evaluated DiNAMO on synthetic and real datasets with two different applications, namely ChIP-seq peaks and Systematic Sequencing Error analysis. DiNAMO proves to compare favorably with other existing methods and is robust to noise.

**Conclusions:**

We shown that DiNAMO software can serve as a tool to search for degenerate motifs in an exact manner using IUPAC models. DiNAMO can be used in scanning mode with sliding windows or in fixed position mode, which makes it suitable for numerous potential applications.

**Availability:**

https://github.com/bonsai-team/DiNAMO.

**Electronic supplementary material:**

The online version of this article (10.1186/s12859-018-2215-1) contains supplementary material, which is available to authorized users.

## Background

Given a set of DNA sequences, the *motif discovery* consists in finding *over-represented* motifs, that are significantly more frequent in the sequences than one would expect by chance. It is a classic task that is nearly as old as bioinformatics and has a large number of applications. The underlying assumption behind this approach is that over-represented motifs indicate a biological function or explain some phenomena. Motif discovery has been extensively used to analyze regulatory regions and detect transcription factor binding sites (TFBS) in promoter sequences of co-regulated genes [1] or to search for enriched motifs in peaks regions for ChIP-seq experiments [2, 3]. Another more recent application is to search for conserved motifs that may induce sequencing errors with next generation sequencing (NGS) instruments [4, 5].

A DNA motif is defined as a short DNA sequence pattern that has some biological significance. Representing a motif with an exact sequence is too rigid and a number of similar words may be combined into a more flexible motif description that allows some variations [6]. Several representations have been introduced in an attempt to characterize these inherent variations. These representations can be divided into two main categories: probabilistic models and word-based expressions. Probabilistic models include frequency matrices, such as Position Weight Matrices (PWMs) or Position Specific Scoring Matrices, and Hidden Markov Models (HMMs). In this context, motif discovery usually relies on local search algorithms, such as Gibbs sampling [7] and expectation maximization (EM) methods, in the widely used MEME algorithm [8, 9]. A main drawback is that these algorithms do not always find the global optimal solution, and that affects their sensitivity [10].

**Fig. 1:**
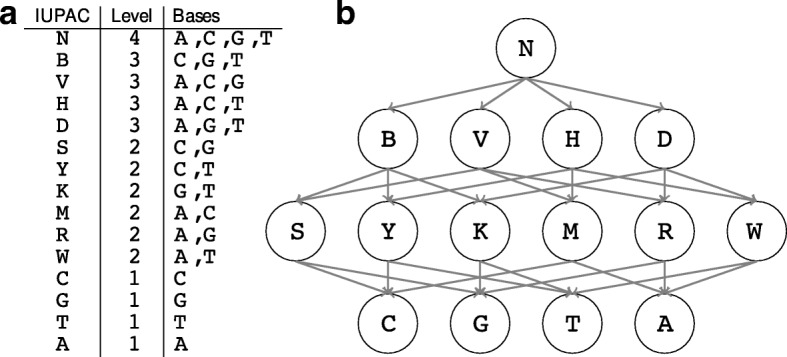
The IUPAC character lattice. **a** definition of the IUPAC alphabet. Each IUPAC character corresponds to a subset of the DNA alphabet {*A*,*C*,*G*,*T*}. The level indicates the degeneracy level of each symbol, which is the cardinal of the subset. **b** the lattice of IUPAC characters constructed from the character lattice

An alternative is offered by word-based representations, that allows to describe a set of words in a combinatorial way. Among the simplest representations may be found the exact strings, like in RSAT [11] and the consensus sequences allowing a few mismatches, like in Weeder [12] and HOMER [13], which are also widely used for Chip-Seq analysis. In this category, we also identify the IUPAC motifs, which use a comprehensive set of wildcard symbols (see Fig. 1a) and have a discriminative power similar to that of probabilistic models [14]. By nature, word-based representations are well-suited for exhaustive enumerative algorithms, which guarantee global optimality, but the bottleneck is the size of the search space. In the case of IUPAC motifs, a naive method cannot be used to search for long motifs because the search space grows exponentially with the motif length. In this perspective, several works, such as YMF [15], MoSDi [16] or Trawler [17], have proposed tractable algorithms at the price of some restrictions on the set of IUPAC motifs, that could be prohibitive depending on the biological application and the size of the genome under consideration.

In this article, we present an exact discriminative method for IUPAC motifs discovery in DNA sequences. With this method, it is possible to efficiently search for weakly represented IUPAC motifs in a large signal dataset, compared to the control dataset, without any restriction on the motif. Our approach is exact because it takes into account all existing exact motifs, whether significant or not, to construct the degenerate motifs. It uses *mutual information (MI)* as an objective function to search for over-represented degenerate motifs. It proceeds in an exact way in a reasonable time, through the use of suitable data structures. The algorithm has been implemented in a software, called DiNAMO, and was evaluated on synthetic datasets as well as on real datasets for two different applications linked to next generation sequencing technologies, namely Chip-Seq analysis and sequence-specific errors detection (SSE).

## Methods

We work on the DNA alphabet {*A*,*C*,*G*,*T*}, and consider the IUPAC alphabet where each character corresponds to a non-empty subset of {*A*,*C*,*G*,*T*}. Thus the IUPAC alphabet has 2^4^−1=15 characters, that are represented in Fig. 1a. We say that a DNA word *w* of length *L*, called an *L-mer*, *matches* an IUPAC motif of the same length if, for each position, the associated nucleotide of *w* belongs to the IUPAC character. A word matching a given IUPAC motif is called an *instance*. For example, the IUPAC motif AWRT has four exact instances {AAAT, AAGT, ATAT, ATGT}.

The DiNAMO algorithm takes as inputs two files in multi-fasta format, containing DNA sequences corresponding respectively to the positive (or signal) dataset $\mathcal {P}$ and the negative (or control) dataset $\mathcal {N}$, and searches for all IUPAC motifs that are over-represented in $\mathcal {P}$ compared to. The algorithm uses three parameters to describe the IUPAC motifs: the length *L*, the number of degenerate letters *d*, which is the maximum number of ambiguous IUPAC characters in the motif (*d*≤*L*), and the *P*-value threshold, which measures the significance of the over-representation.

**Fig. 2:**
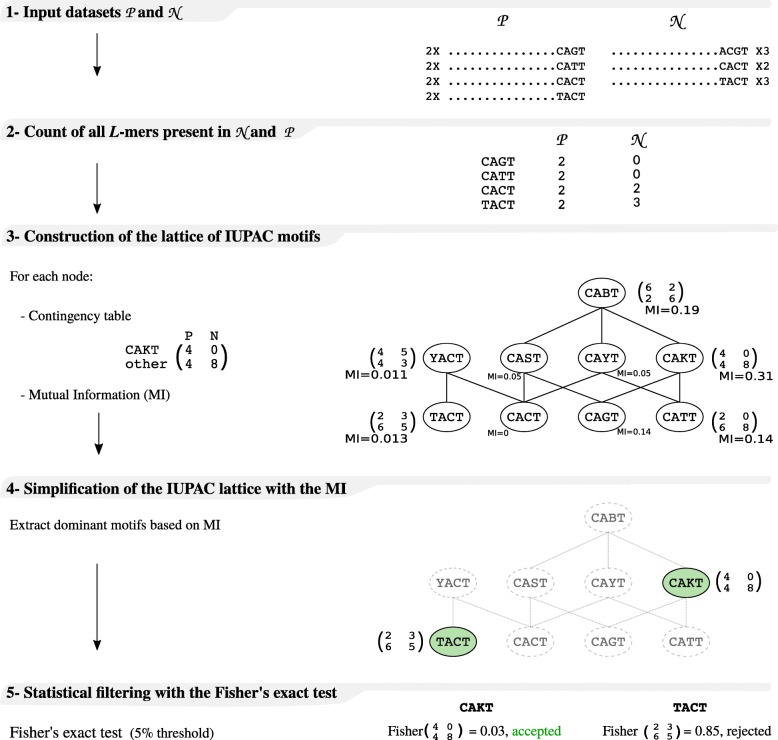
Algorithm of DiNAMO for parameters *L*=4, *d*=4, *p*=0.05 and fixed position mode. The algorithm takes two input files, a positive file, ${\mathcal {P}}$, and a negative file, ${\mathcal {N}}$ (step 1). Here $\mathcal {P}$ and $\mathcal {N}$ both contain 8 sequences. The positive file $\mathcal {P}$ contains 4 different *L*-mers, which numbers of occurrences in $\mathcal {P}$ and $\mathcal {N}$ are stored in a hastable (step 2). The hashtable is used in step 3 to construct the IUPAC lattice. We start from the 4 *L*-mers, and generate IUPAC motifs gradually. The bottom level contains the 4 *L*-mers. Each node at level *i* corresponds to an IUPAC motif for which all instances are present in the initial set of *L*-mers. A link between two nodes of level *i* and *i*+1 indicates that the IUPAC motif at level *i* is a subset of the IUPAC motif of level *i*+1. We do not consider all IUPAC motifs. For example, there is no node for *YAST*, which could have been obtained form the combination of *YACT* and *CAST*, because the instance *TAGT* of *YACT* is not present in $\mathcal {P}$. For each node, we also construct a contingency table using the counts from the hashtable, and we calculate its MI. In step 4, the lattice is simplified in order to keep only the IUPAC motifs that maximize the MI. For example, the MI of *TACT* is higher than the MI of *YACT*, so we remove *YACT*. The final step consists in computing the Fisher’s exact test *P*-value, in order to identify the significantly over-represented motifs

Basically, the algorithm starts from the set of all *L*-mers present in $\mathcal {P}$ and gradually combines these motifs to obtain relevant IUPAC motifs. The main steps of the algorithm are illustrated in Fig. 2.

### Count of all *L*-mers present in $\mathcal {N}$ and $\mathcal {P}$

The first step of the algorithm consists in counting the number of occurrences of each existing *L*-mer in the two input files $\mathcal {P}$ and $\mathcal {N}$. These files can be parsed in two modes: scanning mode, where all windows of length *L* are parsed, one base at a time, or fixed-position mode, where only motifs of length *L* occurring at a specific position in the sequences are taken into account. The choice depends on the application.

The set of resulting *L*-mers present in $\mathcal {P}$ and their associated counts in both $\mathcal {N}$ and $\mathcal {P}$ are stored in a hashtable, which guarantees quick access to the information in the following steps of the algorithm. From this point, only this hashtable is used, and the original sequences are discarded (Fig. 2.2).

### Construction of the lattice of IUPAC motifs

From the hashtable of *L*-mers, we generate all IUPAC motifs of length *L* for which all instances are present in the hashtable. This step is essential because it avoids exploring the space of all the degenerate motifs space. It is performed using a graph, which we call the *lattice of IUPAC motifs*.

As shown in Fig. 1b, the 15 characters of the IUPAC alphabet are naturally ordered by inclusion and form a lattice which has four levels: level 1 for non-ambiguous letters (*A*, *C*, *G* and *T*), which are the bottom elements of the lattice and do not contain any other letters, level 2 for IUPAC symbols combining two non-ambiguous letters (*K*, *M*, *R*, *S*, *W* and *Y*), level 3 for IUPAC symbols combining three non-ambiguous letters (*B*, *D*, *H*, *V*) and level 4 for the letter *N* (*aNy*), which contains all letters and is the top element.

From this character lattice, we define the *lattice of IUPAC motifs* of length *L* for $\mathcal {P}$ as follows. Nodes are IUPAC motifs of length *L* and there is an edge between two motifs *M*_1_ and *M*_2_, if *M*_1_ and *M*_2_ differ at exactly one position, named *i*, and the *i*th letter of *M*_1_ is directly connected to the *i*th letter of *M*_2_ in the IUPAC character lattice. To construct this data structure, we start by building the nodes of all exact *L*-mers present in ${\mathcal {P}}$, that are given by the hashtable. This constitutes the bottom of the lattice. We then gradually generalize each motif by adding one ambiguous character at a time (see Fig. 2.3). To do that, we treat all positions of a given motif by replacing the current nucleotide with an alternative nucleotide. For example, for the word *CACT*, we first look at the first position and test whether *AACT*, *GACT* and *TACT* are present in the hashtable. According to the words found, we generate the corresponding IUPAC motifs. In the example presented in Fig. 2, we generate only *YACT* for this position since only *TACT* is present. Looking at the third position, we generate *CAST*, *CAYT*, *CAKT* and finally *CABT*, since *CAGT* and *CATT* are present. This operation is repeated for each position in the *L*-mer, until the degeneracy threshold *d* is reached. It is done rapidly using the hashtable containing all *L*-mers introduced previously. See Additional file 1, Algorithm 1 for full details on the algorithm. We also compute the total number of occurrences of each IUPAC motif, by summing the counts of all their instances, using the hashtable from the previous step.

At the end of the process, the lattice contains exactly the set of all IUPAC motifs for which all instances are present in $\mathcal {P}$, and each vertex of the IUPAC lattice has a contingency table that contains the counts for the files $\mathcal {P}$ and $\mathcal {N}$.

### Simplification of the IUPAC lattice with Mutual Information

The previous subsection described the method to organize the IUPAC motifs present in the positive dataset $\mathcal {P}$. The next step is to identify motifs that are significantly over-represented in $\mathcal {P}$ in comparison to the negative control dataset $\mathcal {N}$. Different scoring functions have been used in the literature to achieve this task, for example, the Fisher’s exact test *P*-value [18], the Z-score [11], the compound Poisson approximation [16], mutual information [19–21], and many other metrics [22]. Here, we use mutual information (MI) to first explore the lattice and simplify it, and then Fisher’s exact test to rank the selected motifs. The reason is that Fisher’s exact test can be used to determine if a given motif is significantly over-represented in a particular dataset. But the motifs cannot be compared with each other based on their Fisher’s exact test *P*-Value. Indeed, a better *P*-value can be simply due to a larger count. On the contrary, the MI (mutual information) captures the dependency between a motif and a dataset independently from its number of occurrences. The MI of two random variables is a measure of the mutual dependency between the two variables. In our case, the mutual information measures the dependency between the condition and the motif. We use *MI*(*Occurrence*;*Condition*) to compare distinct motifs during the degeneracy procedure. It is defined as follows:

1 $$ MI(O;C) = \sum_{i \in [0,1], j \in [P,N]} p(O_{i},C_{j})\log_{2}\bigg(\frac{p(O_{i},C_{j})}{p(O_{i}) \times p(C_{j})}\bigg)  $$

The random variable *Occurrence* (*O*) corresponds to the absence/presence of the motif *m* (0 for absence, 1 for presence in a sequence) and the random variable *Condition* (*C*) describes the two possible conditions ($\mathcal {P}$ or $\mathcal {N}$).

*Goebel et al.* proposed a method to calculate confidence intervals for the MI [23], based on the non-central Gamma distribution [24]. But comparing such intervals is complicated for further steps of the algorithm, especially for overlapping intervals. In addition, these intervals are obtained by dichotomy method, which is time consuming. So we chose to approximate the MI value from the empirical probabilities obtained from the contingency table (see Table 1). *p*(*O*_*i*_,*C*_*j*_) corresponds to joint probabilities (Eq. 2), where *p*(*O*_*i*_) and *p*(*C*_*j*_) corresponds to marginal ones.

2 $$  p(O_{i}, C_{j}) \approx \frac{\text{\# (\(O_{i}\), \(C_{j}\))}}{ \sum_{i,j} \#\text{(\(O_{i}\), \(C_{j}\))}}  $$

**Table 1:** Contingency table for each motif used for MI calculation

	positive dataset	negative dataset
presence of the motif *m*	a	b
absence of the motif *m*	c	d

*a* (resp. *b*) represents the number of sequences of (resp. $\mathcal {N}$) that contain at least one occurrence of the motif *m*. *c* (resp. *d*) represents the number of other sequences in $\mathcal {P}$ (resp. $\mathcal {N}$). Those four values are used to estimate the joint probabilities *P*(*O*_*i*_,*C*_*j*_) as well as the marginal probabilities *P*(*O*_*i*_) and *P*(*C*_*j*_)

Note that *#*(*O*_*i*_,*C*_*j*_) corresponds to the number of sequences under both conditions *O*_*i*_ and *C*_*j*_.

To avoid redundancy and accelerate the method, we eliminate useless motifs of the lattice that could not improve previously selected motifs, based on their MI. In the lattice, we assume that motif is *dominant* if its MI is greater than the MI of each of its descendants, and that a motif is *dominated* if its MI is smaller than the MI of one of its ancestors. Following these two definitions, we search for all dominant vertices that are not dominated (see Fig. 2.4). To identify such vertices, motifs are sorted by decreasing MI. In this order, the first motif is a dominant vertex that is not dominated. Consequently, we add it to the final list of results, and delete all its descendants and ancestors from the list, as they are dominated. We keep on processing with the next non-deleted motif with maximum MI, until all motifs have been selected or deleted.

### Statistical filtering with the Fisher’s exact test

Finally, we compute the Fisher’s exact test *P*-value for each selected motif. The Holm–Bonferroni method [25] is applied to adjust the *P*-values and counteract the problem of multiple comparisons. We only keep motifs with a *P*-value below the *P*-value threshold (Fig. 2.5). See Additional file 1, Algorithm 2.

### Detection of secondary motifs

Secondary motifs are IUPAC motifs for which the *P*-value is lower than the threshold, while being not optimal and having no instances in common with previously detected motifs with better *P*-value. Usually, such motifs are detected by masking all the instances of the motif found in the sequences, and by re-runing the whole algorithm. In our algorithm, we use our lattice representation, and mask all the instances directly in the lattice, by eliminating the ancestors of the descendants of the found motif. This allows a better runtime.

### Overlapping motifs

**Fig. 3:**
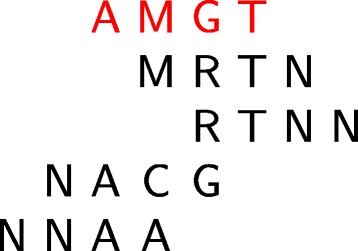
Clustering of overlapping motifs. The reference motif is *AMGT* (in red). Motif length is 4, so the minimum overlap between this motif and all other motifs of the cluster is $\frac {4}{2}=2$. The motif *MRTN* matches with *AMGT* because the letter *G* is included in *R*. Likewise, the motifs *NACG* and *NNAA* matches with *AMGT* because the letters *A* and *C* are both included in *M*

In the scanning mode, the algorithm has to take into account overlapping motifs. For example, if the motif *AMGT* is over-represented in the dataset, then *MGTN*, *GTNN*, *NAMG* that all overlap with *AMGT* could also be detected as over-represented motifs. In order to avoid this and return only the representative motifs, an optional post-processing is added, which consists in clustering the over-represented motifs based on their sequence similarity. The motif with the highest MI is first selected as a reference motif, and all other motifs are aligned against it, with one position shift at a time. In this alignment, we consider that two IUPAC symbols can match, if they are identical or included in each others. In order to avoid clustering too many motifs in the case of datasets of low complexity, we do not allow the extension of the main motifs to more than half of their size on either side (Fig. 3). This greedy method is close to the clustering method used by RSAT - pattern-assembly tool [11], with the difference that we take into account the IUPAC alphabet inclusions.

### Implementation

DiNAMO is implemented in C ++ using the libraries Sparsepp [26] and boost [27]. It is freely available under the GNU Affero General Public License, version 3. It can be easily installed (binaries for different Linux, MacOS and Windows are available) and used on a desktop machine (< 8 GB of RAM, see Table 3).

## Results and discussion

We applied DiNAMO on multiple datasets. The first one is a synthetic dataset with implanted motifs. The two others are empirical case studies corresponding respectively to peak sequences for ChIP-Seq application and to genomic regions prone to systematic sequencing errors. We also compared DiNAMO with three other programs, MEME-CHIP [28], HOMER [13] and Discrover [19], that use different models for motif representation. MEME-CHIP is from the MEME suite and runs two motif discovery algorithms, MEME [29] and DREME [18]. The MEME algorithm uses expectation maximization to discover motifs modeled by PWMs. DREME uses regular expressions together with the Fisher exact test *P*-Value. Discrover is based on HMMs and uses the Baum–Welch training algorithm. In contrast, HOMER uses a simple mismatch model and the hypergeometric distribution to score the enrichment of oligos. For Discrover we had to specify as a parameter the number of motifs to search for. We fixed this value to 10. For HOMER, we keep the default parameter (-n=25). We use the same length of motifs for each tool, with the default parameters.

### Evaluation on synthetic datasets

In this experiment, we constructed a series of simulated datasets that allowed us to control the number, frequencies, and global level of degeneracy of the over-represented motifs. This setting also allowed us to measure the sensitivity and specificity of the tools, since we know exactly which motifs have been implanted.

**Generation of random sets of IUPAC motifs.** We constructed several sets of IUPAC motifs of fixed length 6 to implant them in the positive dataset, with the following varying parameters: the number of motifs in the set and the *IUPAC content* of the motif. The number of motifs ranges from 1 to 4. The *IUPAC content* is calculated by summing up the degeneracy level of all the motif letters (see Fig. 1a). The allowed values are 6,8,10,12 and 14. The lowest value 6 corresponds to exact motifs with no degenerate characters, while the largest value 14 corresponds to motifs such as ANANAH, MRNWYY or BAVCHB. We considered all possible combinations of those two parameters, which gave a total of 20 combinations. For each combination, we generated 5 sets of motifs, giving rise to **100** different sets of motifs (Additional file 1, Table S1).

**Implantation of the random IUPAC motifs.** Two files of 5000 random DNA sequences were built with the RSAT sequences generator [11] using independent and equiprobable nucleotide distribution. These two files are used for each set of motifs. The first one serves as a negative control dataset. The second one is for the positive signal dataset, in which we implanted motifs at **6** different frequencies: 5%, 4%, 3%, 2%, 1% and 0.5% of the number of initial sequences. Each IUPAC motif is uniformly represented by its instances, and all motifs in the set are implanted with the same frequency. We repeated this operation **100** times per set of motifs.

At the end, we evaluated the four different tools on 100×6×100=60,000 different datasets.

**Fig. 4:**
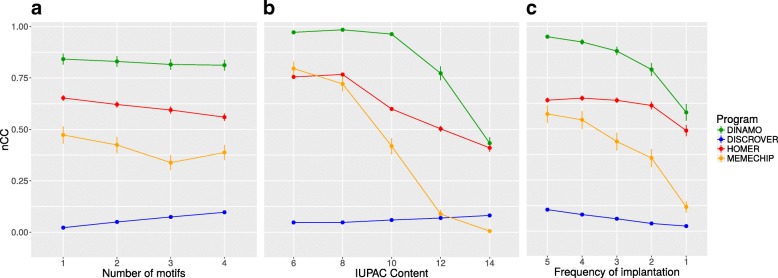
The impact of each simulation parameter on the nCC value of the 4 compared programs. In each graph, a single parameter is varied while all the others are enumerated and combined. **a** Number of motifs parameter, **b** IUPAC content parameter, **c** Frequency of implantation

**Results** We used the *nucleotide level correlation coefficient* (nCC) to evaluate the performance quality [30]. The nCC is a balanced measure that captures the sensitivity and the specificity of the predictive method.

3 $$  {nCC} = \frac{TP \times TN - FN \times FP}{\sqrt{(TP + FN)(TN + FP)(TP + FP)(TN + FN)}}  $$

where *TP/TN/FP/FN* are the number of nucleotides in the dataset that are estimated to be true positives/true negatives/false positives/false negatives. The nCC takes on values between 1 (perfect prediction) and -1 (perfect inverse prediction). An nCC equal to 0 means that there is no correlation between the prediction and the actual occurrences of the motifs. To summarize the results of the repeated experiences for each set of parameters, we calculated the average nCC. Results are reported in Fig. 4.

DiNAMO achieves the best results for the detection of degenerate motifs compared to all other tools (best nCC value). Discrover has the weakest nCC value. MEME-CHIP achieves good results with exact motifs (*IUPAC content* equal to 6 in Fig. 4b), but its nCC value falls quickly with the growing *IUPAC content* of the motifs. HOMER achieves nCC values which are the closest to DiNAMO values. However, the motifs that are identified are nearly exact and weakly degenerate. Thus, instead of finding a single degenerate motif, HOMER reports multiple exact instances representing the same motif. That is why its nCC value falls with the increasing number of implanted motifs, in contrast to DiNAMO (Fig. 4c).

In Additional file 1, Figure S2, we provided more details about the impact of each parameter at variable frequencies on tools performance.

We also evaluated the specificity of each tool with 100 randomly generated datasets without any implanted motifs. The specificity (SPC) measures the proportion of identified true negatives in the dataset (*SPC*=*TN*/*N*, where *N* corresponds to the total number of nucleotides in the dataset). As for implanted motifs tests, we ran the tools with the default parameters, and we searched for motifs of length 6 with up to 6 degenerate positions. Results shows that all tools have a high specificity (> 0.98) (see Additional file 1 Figure S3).

### ChIP-Seq datasets

A main application of motif discovery is to extract DNA binding site motifs from peaks obtained from ChIP-seq data. We used the five datasets introduced in [18] for the evaluation of their tool DREME: three mouse embryonic stem cell datasets [31], corresponding respectively to the transcription factors Oct4, STAT3 and Sox2, and two mouse erythrocyte datasets [32, 33], corresponding respectively to Gata1 and Klf1. The number of peak sequences in each dataset is reported in Table 2.

**Table 2:** Number of peak sequences for each of the five ChIP-seq datasets

Dataset	Number of sequences
GATA1	14,351
SOX2	4526
OCT4	3761
STAT3	2546
KLF1	945

For each of those five datasets, the positive file was constructed by extracting sequences of 100 bp length centered around each peak, and the negative dataset by shuffling these sequences with the dinucleotide shuffling tool from the MEME suite [8]. We ran DiNAMO, MEME-CHIP, HOMER and Discrover with IUPAC motifs of length *L*=7 containing up to 3 degenerate letters (*d*=3). Like all the TFBS (Transcription Factor Binding Site) discovery tools, we used the sliding window mode in DINAMO and screened each position of the sequences. Then, we applied the clustering procedure described in Section *Overlapping motifs*.

Predicted IUPAC motifs are then identified by comparing them against the frequency matrices of the JASPAR database [34], using the TOMTOM tool of the MEME suite [8]. Since each motif can match multiple frequency matrices in JASPAR, we considered the ten first significant matches of TOMTOM.

All methods performed well on the full dataset (100%), and correctly identified the expected transcription factor. Moreover, they found several secondary motifs. For each dataset (GATA1, SOX2, OCT4, STAT3, KLF1 respectively), 18,17,13,17,5 cofactors are found by DiNAMO, 11,16,10,3,2 by MEME-CHIP, 9,10,12,7,6 by HOMER and 3,4,4,2,2 by Discrover. Most of these motifs have been already validated experimentally as co-factors of the principal transcription factor (see Table S2 in the Additional file 2).

**Fig. 5:**
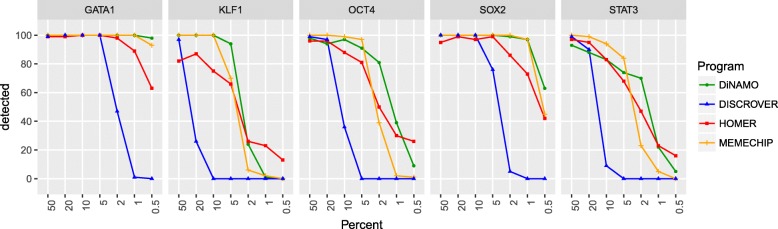
Graph showing the detection sensitivity of the studied motif. For each dataset, we do a sampling with different sizes. The x-axis shows the amount of token sequence from the original file. For each percentage the sampling experience was repeated 100 times. The y-axis represents the number of times the motif was detected among the 100 repetitions

Each positive dataset was then downsampled in order to evaluate the performance of the methods according to the sample size. We performed seven different sample sizes: 50%, 20%, 10%, 5%, 2%, 1%, 0.5% of original peak files. For each sample size, the experiment was repeated 100 times, and we counted the number of times that the expected motif, corresponding to the transcription factor binding site, was correctly found (Fig. 5).

We noticed that DiNAMO and MEME-CHIP predict the true TF motif in most cases (nearly 100% of cases until 5% of peak sequences), but DiNAMO has a better sensitivity with low frequencies. It is important to notice that in fact, MEME-CHIP use two tools (MEME & DREME) to achieve such sensitivity, which also affects the program running time (Table 3).

**Table 3:** Running time and memory requirement

	CPU (sec)	RAM (MB)
GATA1		
DiNAMO	100	3400
MEMECHIP	1280	70
HOMER	18	555
Discrover	170	244
FIXED		
DiNAMO	0.75	41
MEMECHIP	1.78	30
HOMER	0.88	120
Discrover	192	141

The table on the left shows the GATA1 ChIP-seq dataset (14,351 sequences of length 100, scanning mode). The table on the right shows the synthetic dataset (5000 sequences, fixed position mode). These values were achieved on one CPU Intel Xeon 1,96GHZ

HOMER also achieves good results, but it is less sensitive than MEME-CHIP and DiNAMO (the difference of TFBS motif detection proportion reaches approximately 20%) and the HOMER’s curve is inexplicably reversed for the smallest datasets (0.5% of peak files). DISCROVER has the weakest sensitivity, probably due to the HMM model.

### Systematic Sequencing Errors

Next generation sequencing technologies are characterized by their high throughput compared to the Sanger method [35]. The main drawback of these technologies is their error rate, that varies from 0.001% to more than 1% depending on the technology [36]. To overcome these errors, the variant callers use statistical filters, that require principally a high depth of coverage to call variants and filter sequencing errors.

The task remains difficult when searching variants with low allelic fraction [37, 38]. These variants correspond to clonal or sub-clonal mutations, that can be found in heterogeneous cancer samples for example [39]. To detect such variants, we need a highly sensitive and specific analysis. For the sensitivity of mutation calling method, we usually use low thresholds to call a mutation [38]. On the other hand, to obtain a good specificity, we sequence the interesting regions with a high coverage (≥ 100×) to be able to separate more easily low allelic variants from sequencing errors.

However, high-coverage sequencing does not eliminate all errors, especially the systematic sequencing errors, which are a major issue in high-throughput sequencing platforms [40]. On the contrary, it has been shown that the number of systematic errors that are called as true mutations (False positive rate) increases with sequencing coverage [41, 42]. This errors occur mostly at specific positions in the reads and can be confused with true genomic variations.

In [43], the authors showed that systematic errors depend on the upstream context and that there are over-represented motifs upstream of the sequencing error positions in Illumina reads, in particular *GGT* motif. This observation was also corroborated in [4], where the authors applied a motif discovery approach and found a similar motif. This last approach, however, did not take into account the full IUPAC alphabet. It was limited to exact motifs with only N letters, and not adapted for large genomes, like the human one. GATK [44] integrates the motifs information also by recalibrating the bases quality score in the BQSR module, but their method is limited to exact motifs of length 2 for mismatches and 3 for indels.

We applied DiNAMO to analyze in a more comprehensive way the upstream regions of systematic sequencing errors. For that, we used the monocyte dataset described in [43]. This compilation contains 3272 genomic coordinates of sequencing errors on the Human genome (Hg18). We kept only the positions not located in chromosomal extremities (3249 positions). For each of them, we retrieved a window of length 42 (the error position and the 41 upstream nucleotides) on the two genome strands, giving a total of 6498 sequences. These sequences were then split into two equal fragments of length 21. Sequences that contain the error position constituted the positive dataset, while the other sequences constituted the negative dataset. In this way, we made sure of having the same nucleotide frequencies to withdraw potential bias due to codon usage.

DiNAMO was first launched on these two sets of sequences to search for motifs of length *L*=6 with at most *d*=6 degenerate letters, at the last position of the extracted windows. The first motif found by DiNAMO is *NNRGGT* (adjusted *P*-value < 10^−324^), which confirms the initially reported motif *GGT* and gives a more precise picture of the involved motifs at the same time. This motif shows that *GGT* in fact extends to *RGGT*. DiNAMO also found SBTGGW and NBGGGA. These motifs show that *GGA* could induce systematic errors, which is also reported in [4].

Finally, we launched DiNAMO on the other positions of the sequences, to search for potential alternative contexts located at a greater distance from the sequencing error. No significant motifs were found, showing the selectivity of the algorithm.

## Conclusion

In this article, we presented an exact algorithm for IUPAC motif discovery, which achieved excellent results on different types of biological applications. In all cases, DiNAMO was able to detect subtle signals with high sensitivity. The method successfully explores all degeneracy levels of the IUPAC alphabet and does not require any prior knowledge, other than the length of the motif and the maximal number of degenerate positions in the motif. The second major advantage of the method is that it is tractable in practice. In Table 3, we report the execution time and the memory space required to run DiNAMO. Due to the data structures involved, it needs significantly more memory space, but can still be run on a desktop computer. All these characteristics make DiNAMO an efficient and universal algorithm that is well-suited for any type of applications.

## Additional files

Additional file 1 Supplementary materials. **Algorithm 1**, **Algorithm 2**, **Figures S1,S2** and **Table S1**. (PDF 431 kb)

Additional file 2 Predicted cofactors. **Table S2**. The complete table of predicted cofactors on each dataset with the three compared software. (PDF 60 kb)

Additional file 3 Raw predicted cofactors interaction graphs from Ingenuity Pathway Analysis (IPA). Files with ’_high’ suffix (for high confidence) represent data from “Ingenuity expert findings” and “Experimentally observed” databases. Files with ’_low’ suffix (for low confidence), represent data from all IPA databases. (ZIP 2519 kb)

## Abbreviations

EM: Expectation Maximization
HMM: Hidden Markov Model
MI: Mutual Information
nCC: Nucleotide level Correlation Coefficient
NGS: Next Generation Sequencing
PWM: Position Weight Matrice
SSE: Sequence Specific Errors
TFBS: Transcription Factor Binding Sites
