# Supplementary material for: DiNAMO: highly sensitive DNA motif discovery in high-throughput sequencing data
Source: BMC Bioinformatics. 2018 Jun 11;19:223. doi: 10.1186/s12859-018-2215-1 (PMC5996464; doi:10.1186/s12859-018-2215-1)
Supplement: Supplementary file 1 — Supplementary materials. Algorithm 1, Algorithm 2, Figures S1,S2 and Table S1. (PDF 431 kb) [file 12859_2018_2215_MOESM1_ESM.pdf]

Supplementary materiel

1 Algorithms

The algorithm 1 describes the process of degeneration of an initial set of exact motifs P, with a degeneracy parameter d, that corresponds to the maximal allowed number of degenerate positions. Each motif m in the set P is scanned position by position. We treat all positions of the current motif m by replacing the current nucleotide with all the accepted IUPAC symbols. For example, for the word AA present in P, we first look at the first position $i = 1$ and test whether CA, TA and GA are present in P (Algorithm 1, line 30). According to the other words found in P (for example TA and GA), we generate the corresponding IUPAC motifs among WA, RA, KA and DA (line 31). The set of degenerate motifs P', obtained from each position of each original motif in P (line 35), is then reused to generate motifs with more additional degenerate IUPAC letters, and the process is repeated until the degeneracy parameter d is reached.

Algorithm 1: degeneration of a set of motifs P.

Algorithm 1 Degenerate set of motifs

```
function SearchForNeighbors(m, i, P)
    Input :
        • m: the motif (string in IUPAC alphabet)
        • i: position of m to degenerate (integer)
        • P: set of motifs
    Output : Neighbors: set of motifs                    ▷ neighbors of m
    Neighbors ← ∅
    for each l ∈ {A, C, G, T} do
        m[i] ← l
        if  m ⊂ P then
            Neighbors ← Neighbors ∪ m
        end if
    end for
    return Neighbors
end function

function Combine(Neighbors)
    Input : Neighbors: set of motifs             ▷ neighbors of to combine
    Output : Neighbors: set of possible IUPAC motifs
    Possible_IUPAC_motifs ← ∅
▷ generate possible IUPAC combinations from the set Neighbors
▷ example: AA, TA, GA → WA, RA, KA, DA
    return Possible_IUPAC_motifs
end function

function Degenerate(P, d)
    Input :
        • P: set of exact motifs to degenerate
        • d: degeneracy level (integer)
    Output : All_degenerate_motifs      ▷ set of degenerate motifs, obtained
from P
    All_degenerate_motifs ← ∅
    degeneracy_level ← 0
    while degeneracy_level < d do
        P′ ← ∅
        for each motif m ∈ P do
            for each position i ∈ [1..|m|] do
                Neighbors ← SearchForNeighbors(m, i, P)
                DegenerateMotifs ← Combine(Neighbors)
                P′ ← P′ ∪ DegenerateMotifs
            end for
        end for
        P ← P′
        All_degenerate_motifs ← All_degenerate_motifs ∪ P′
        degeneracy_level ← degeneracy_level + 1
    end while
    return All_degenerate_motifs
end function
```

Once we obtain the list of all possible degenerate motifs from algorithm 1, the lattice is constructed by connecting with a directed edge the motifs that differ at exactly one position with an inclusion rule (see chapter Methods: Construction of the lattice of IUPAC motifs). The next step consists of searching for *dominant* motifs (motifs with maximal mutual information). The function $MaxMiNode$ (algorithm 2, line 6) returns the motif (node) having the maximum mutual information (*MaxMI*) value in the lattice. As illustrated in the figure S1, its ancestors and descendants are then deleted, since they are *dominated* (lines 7 and 8). We delete also all the ancestors of any descendant of the maximal mutual information node, that corresponds to redundant motifs (lines 9).

The P-Value of retained motifs is calculated with the Fisher's exact test, using the contingency table associated to each node in the lattice (Fig. 2.5).

Algorithm 2 Simplify lattice

```
 1: function SimplifyLattice(G, pValue=0.05)
 2:     Input : G: lattice of degenerate IUPAC motifs
 3:     Output : Final_motifs: set of significantly over-represented motifs
 4:     Final_motifs ← ∅
 5:     while G ≠ ∅ do
 6:         max_node ← MaxMiNode(G)                          ▷ See figure S1
 7:         DeleteAncestors(max_node, G)
 8:         DeleteDescendants(max_node, G)
 9:         DeleteAncestorsOfDescendants(max_node, G)
10:         if FisherExactTest_Pvalue(max_node) < pValue then
11:             Final_motifs ← Final_motifs ∪ max_node
12:         end if
13:         Delete(max_node, G)
14:     end while
15:     return Final_motifs
16: end function
```

Algorithm 2: Algorithm to simplify a lattice of degenerate motifs G.

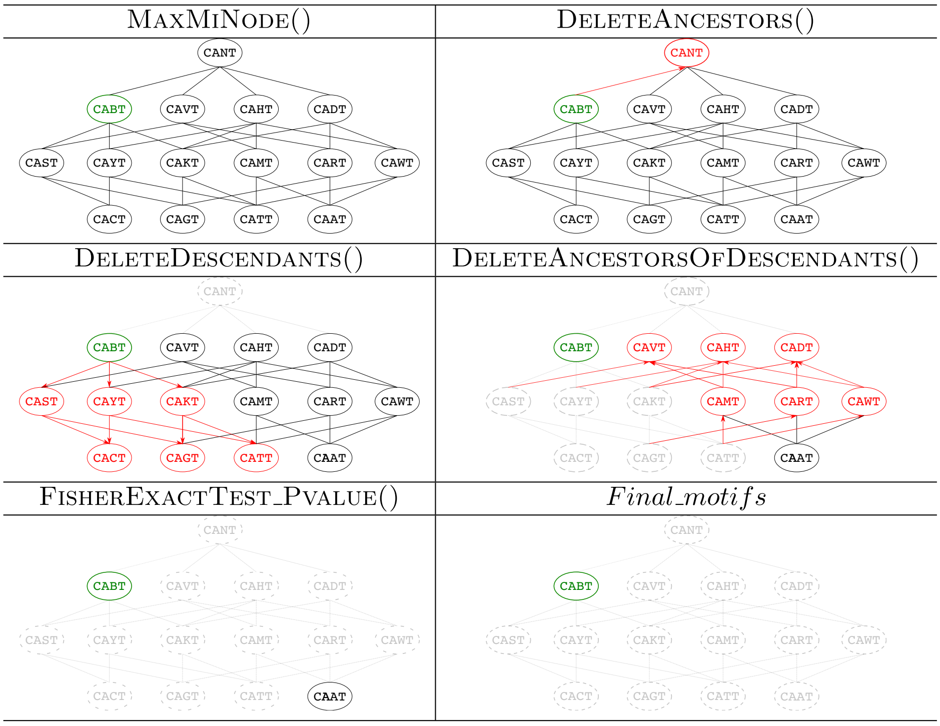

Figure S 1: Graphical example of impacted nodes for each function of the algorithm 2.

2 Evaluation on synthetic datasets

In this experiment, we study the impact of each of the two parameters, respectively, *the IUPAC content* (see chapter *Results*, paragraph *Generation of random sets of IUPAC motifs*) and *the number of implanted motifs* at variable frequencies of implantation on the motif discovery quality. According to the Figure S2, we can notice that for DiNAMO the nCC value does not vary much with the IUPAC content between 6 and 12 (a). For MEME-chip, the nCC value is good for exact motifs (IUPAC content = 6), while the discovery precision of highly degenerate motifs (high *IUPAC content*), decreases sharply with the frequency of motifs implantation (b). In other words, it is difficult for MEME-CHIP to detect degenerate motifs at low frequencies. For HOMER, we notice that the nCC value decrease proportionally to the IUPAC content. This means that HOMER is also sensitive to the IUPAC content, but less sensitive then MEME-CHIP. For Discrover, we can notice that the frequency parameter is the most impacting parameter, because regardless the value of the IUPAC content parameter, the nCC value is low and increasing with the frequency (d). We can also notice that the number of implanted motifs does not affect the nCC value of the four studied software. For each tool, the four curves have the same shape (e,f,g,h).

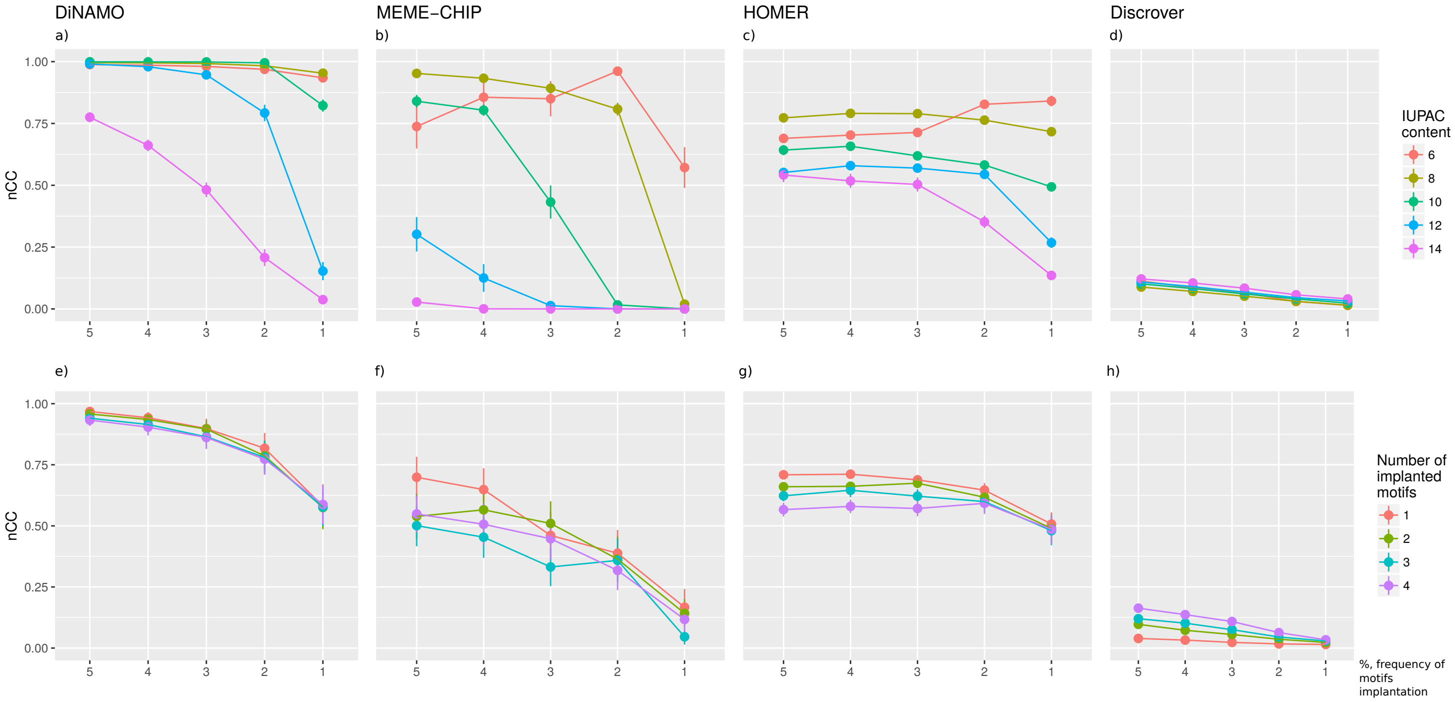

Figure S 2: Impact of each parameter on the motif detection quality at each frequency of motifs implantation.

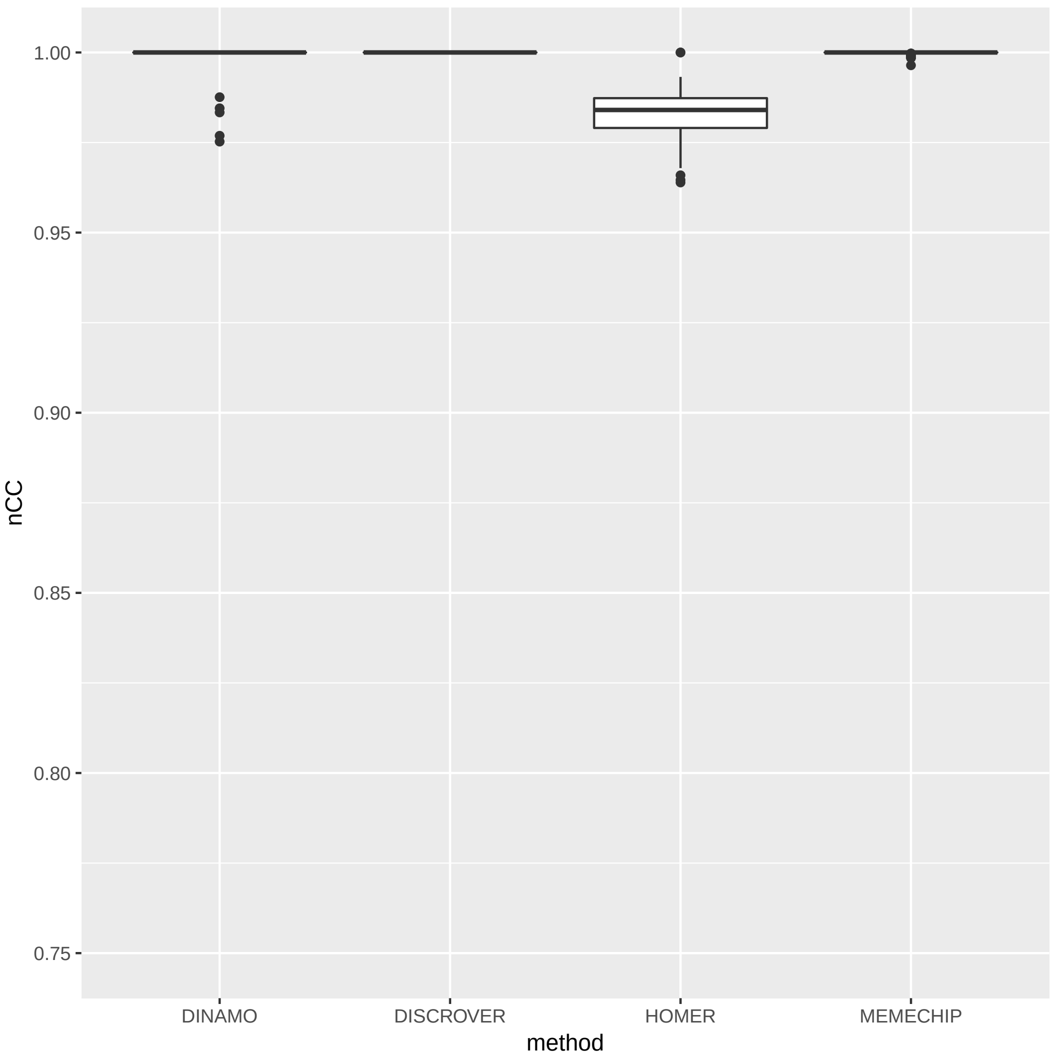

Figure S 3: The specificity of each tool calculated on random datasets. 100 random datasets that do not contain any implanted motif are generated (Bernoulli distribution) in order to evaluate the False positive rate (5000 random sequences for each dataset). As for implanted motifs tests (section Evaluation on synthetic datasets), we ran the tools with the default parameters, and we searched for motifs of length 6 with up to 6 degenerated positions. Results show that all tools have a high specificity (> 0.98).

Table S1: Implanted motifs in synthetic datasets: the 100 randomly generated sets of motifs. Multiple motifs are separated by '_', when implanted in the same dataset.

	IUPAC content	6	8	10	12	14
Number of implanted motifs						
1		GCCGAT	CTGVCG	CNWCGG	YBATNT	HWWHCB
		AAGAGT	AWACRA	SCWSKT	SNTTKK	GRMNHS
		CCTCAG	RACCTS	KMTCYS	VCAHAH	YVBDTM
		AAAAGC	RTGCGS	TKSRKA	DSAYRY	BRCNYW
		CTTTGC	GMRTCT	RKCTDA	NSCGKS	TVSDNA
2		GATTTC,TGAGGG	MGTKAC,RCGCGK	GYTGWB,MTWASW	RNSTCW,TRRWHR	RHMYDK,GYBMNR
		AGCCTT,ACGTCG	CTVTAG,AYMTCA	BKTRTG,KCTYYW	WYRHTM,SCSDBG	TNHSKK,KHHHAS
		TTAGGT,TTTTTT	CDCCTA,MTGGCS	HAYTMA,RTBGMA	DKYADG,GHRRBT	BNCRRY,CNGSHH
		CAGCTC,GCAGCG	ACMTCR,DGTACT	HGAYTS,KVSCAT	SAVWRR,CYTDBM	RYRRVB,CSWMVN
		ATATTT,TTCAGT	ATYCRT,CCMTYC	AKYTWM,BKWTAT	MSADRY,MVRWTS	DDDDTG,DYVDCY
3		GGTGCG,AGTGGC,TTAGGT	TTRCCK,TCTGBC,GAGGSW	ACCRBY,ARKCRS,GKRKRC	CVSCGN,BSTGBR,NWWATS	MVKSTN,MADDMH,BYWHRK
		TTCCGT,CGATGT,GGGCAC	GCMTSG,TCTCSS,AACWWC	BCSTCM,TWCSMK,CRCBKG	DRCKDG,ATTNWD,RHTAHS	BGKYNS,SNWRSM,HBRMGD
		ATGGTG,CCGTGT,ACTGTT	KACCAM,TAGWRG,AWWGCT	AKKWWC,TKASKK,ASWWCS	DSSVCC,RBKTKS,BKCARB	YDMVSY,HBVAWK,BMRMDS
		GGATTT,GAAAGT,TTACTA	ASCKAC,TDTTCC,RCCCSG	AYBAYC,RYSCMG,TGYCSH	MTHKWK,HCGBMR,HNCRCC	DSHSVC,BHHARY,AHSWHH
		TGAACT,TGTACC,TCTCCT	CGTSAS,AGGAHC,CYATSA	YTYAYR,VMRCTG,CCYMVG	YWACHB,WSBMYA,MMDMKT	HKWBBT,SVTSNK,WCSNDY
4		CCGCTG,CGGTTC,TTCGCC,AAACGC	TTGMCW,YAAAST,AVCACA,RCTCTK	HMGWAG,MCCDYA,SRYRTC,AWRHCA	GWHWYK,YBWDCT,TBMKYK,AVACWN	GABDVH,KKHWRH,WANYHM,MMBVKK
		CTAAGA,CATGCG,AGTGGC,GCACAG	AGGKST,MACGYG,GKWTCT,GCAMAK	CBTMAY,RAWHTT,VSATAM,CHHGGT	HRMSWA,YYYYRK,SHKWGW,WSHKGK	HBCKBW,KSTDKN,KWBWYB,HBKGMB
		GCCGCT,GCCCCC,CATTGT,TGTTGA	ACRGAS,TWTSGC,AMTAGY,SWGCGC	YTBTYC,RAMAKK,TTSWKK,CGTTWN	TDWSRW,SGYVVG,SGVKWY,SRBRGW	MAHDDM,MVHWDT,YMABVB,THDVSW
		ATTGCT,GCAGCT,GGAACC,CCCAAG	AMYGCC,TKTGGS,TGAMTW,WGRCTT	MKRYCG,KGAWKM,YYSCTS,RCAKHC	HSDCSC,YWKSDG,STDBCR,NYCGWK	HSDRYS,DMMSRB,WMKCNV,HWDHGY
		ACCAAA,GATAGG,CAGTTC,CTCGTC	TCKWGC,MCSTCG,AATYAR,TKWCCA	VRGCCS,MCMMKC,BYGKGG,WYAAMM	CTSRRN,NWAGCD,RGVMRS,YMWMTH	BVSBGW,TWSHMN,VWTYVD,NRCDBT
