# Supplementary material for: DiNAMO: highly sensitive DNA motif discovery in high-throughput sequencing data
Source: BMC Bioinformatics. 2018 Jun 11;19:223. doi: 10.1186/s12859-018-2215-1 (PMC5996464; doi:10.1186/s12859-018-2215-1)
Supplement: Supplementary file 2 — Predicted cofactors. Table S2. The complete table of predicted cofactors on each dataset with the three compared software. (PDF 60 kb) [file 12859_2018_2215_MOESM2_ESM.pdf]

# Predicted cofactors

| | DINAMO | MEMECHIP | DISCROVER | HOMER |
|---|---|---|---|---|
| GATA1 | STAT1 | STAT1 | KLF1 | MYC |
| | OTX1 | KLF1 | STAT1 | KLF4 |
| | MAX | RUNX1 | RUNX1 | SPI1 |
| | DUXA | OTX2 | | STAT1 |
| | SP1 | Ahr::Arnt | | ID2 |
| | Myog | Myog | | OTX2 |
| | TAL1::TCF3 | KLF5 | | TGIF1 |
| | Hmx3 | FOXP2 | | MTF1 |
| | PROX1 | FLI1 | | HOXB13 |
| | YY1 | YY1 | | |
| | FOSL1 | FOSL1 | | |
| | Ahr::Arnt | | | |
| | ELK4 | | | |
| | MTF1 | | | |
| | XBP1 | | | |
| | MYC | | | |
| | SREBF1 | | | |
| | CENPB | | | |

| | | | | |
|---|---|---|---|---|
| SOX2 | POU3F4 | POU3F4 | POU3F4 | Pou2f3 |
| | KLF5 | POU4F2 | ZIC1 | E2F4 |
| | mix-a | KLF5 | KLF5 | EGR1 |
| | Nr5a2 | ZIC1 | POU4F2 | ELK4 |
| | SCRT2 | ESRRB | | FOXO3 |
| | USF2 | EWSR1-FLI1 | | mix-a |
| | PROP1 | TEF | | ESRRB |
| | BHLHE22 | Klf1 | | Tcf7 |
| | TEAD3 | mix-a | | Pparg::Rxra |
| | CDX2 | SCRT1 | | HES7 |
| | STAT1 | E2F4 | | |
| | POU3F2 | TEAD3 | | |
| | FOXO3 | POU2F2 | | |
| | NRF1 | SOX8 | | |
| | NR1H2::RXRA | ETV2 | | |
| | BCL6B | LIN54 | | |
| | ELF4 | | | |

| | DINAMO | MEMECHIP | DISCROVER | HOMER |
|---|---|---|---|---|
| OCT4 | SPIC | POU3F4 | Sox6 | POU2F2 |
| | POU3F4 | POU4F2 | Pou2f3 | Sox17 |
| | KLF5 | Sox6 | KLF5 | POU3F4 |
| | NR4A2 | KLF5 | SP1 | ELK1 |
| | MAFG::NFE2L1 | SPIC | | GATA1::TAL1 |
| | ETV6 | ESRRB | | SP1 |
| | ZIC1 | ELK4 | | ESRRB |
| | FOXG1 | TCF4 | | Klf1 |
| | MEF2C | LIN54 | | SP4 |
| | MAFK | BCL6B | | Creb5 |
| | GSC | | | TEAD3 |
| | ESRRB | | | ID2 |
| | RELA | | | |

| | | | | |
|---|---|---|---|---|
| STAT3 | NR4A2 | KLF1 | GATA2 | SP3 |
| | Ascl2 | GATA2 | KLF1 | GATA2 |
| | KLF4 | KLF13 | | KLF5 |
| | ESRRG | | | OTX2 |
| | SP1 | | | TFAP4 |
| | GSC | | | FOXH1 |
| | ID2 | | | NFAT5 |
| | BCL6B | | | |
| | POU6F2 | | | |
| | FOSL1 | | | |
| | POU4F1 | | | |
| | TBX15 | | | |
| | ELK1 | | | |
| | GLIS1 | | | |
| | TCF7L2 | | | |
| | SRF | | | |
| | ELF3 | | | |

| | | | | |
|---|---|---|---|---|
| KLF1 | GATA1/2 | GATA1/2 | GATA1/2 | SP3 |
| | Tcf12 | KLF13 | KLF16 | GATA1/2 |
| | Dmbx1 | | | OTX2 |
| | E2F7 | | | TFAP4 |
| | AR | | | FOXH1 |
| | | | | NFAT5 |

**Table S2: Comparison of detected cofactor motifs using the 4 different programs.**

Motifs are sorted by their score given by each program. Annotation is performed by the TOMTOM tool and Jaspar database.
For motifs that match to multiple motifs in Jaspar database, we report in this table and we test in Ingenuity Pathway Analysis just the first one .
The predicted cofactors are colored in green if there is a "protein-protein/DNA" interaction with the main TF, described in the "Ingenuity expert findings" and "Experimentally observed" data . Cofactors in yellow are found using all INGENUITY databases.

*IPA: INGENUITY pathway analysis, Build: 463341M, content: 42012434
