# Supplementary figures and images for: DiNAMO: highly sensitive DNA motif discovery in high-throughput sequencing data
Source: BMC Bioinformatics. 2018 Jun 11;19:223. doi: 10.1186/s12859-018-2215-1 (PMC5996464; doi:10.1186/s12859-018-2215-1)

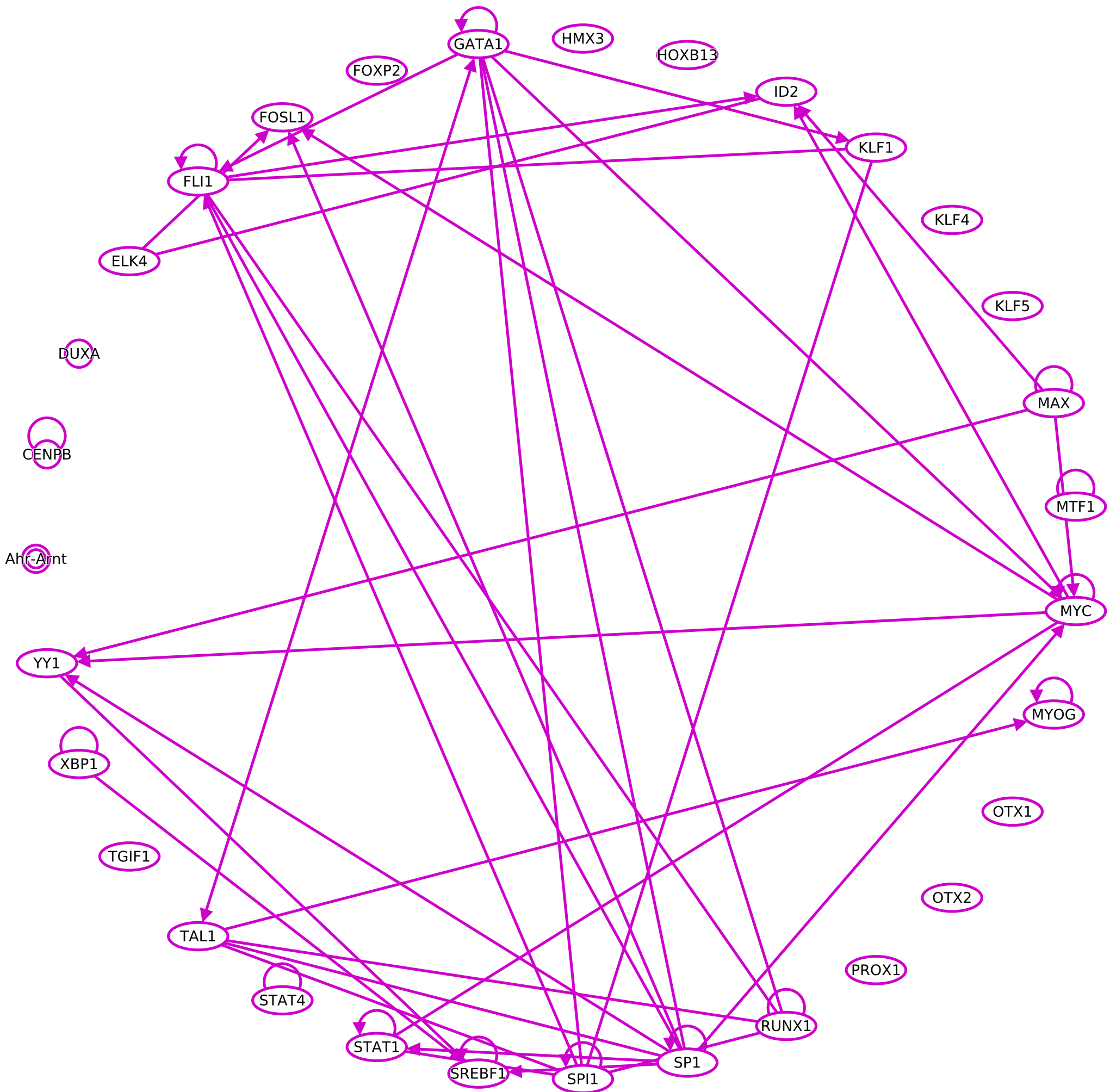

Supplement: Supplementary file 3 — Raw predicted cofactors interaction graphs from Ingenuity Pathway Analysis (IPA). Files with ’_high’ suffix (for high confidence) represent data from “Ingenuity expert findings” and “Experimentally observed” databases. Files with ’_low’ suffix (for low confidence), represent data from all IPA databases. (ZIP 2519 kb) [file 12859_2018_2215_MOESM3_ESM.zip › GATA1_high.pdf]

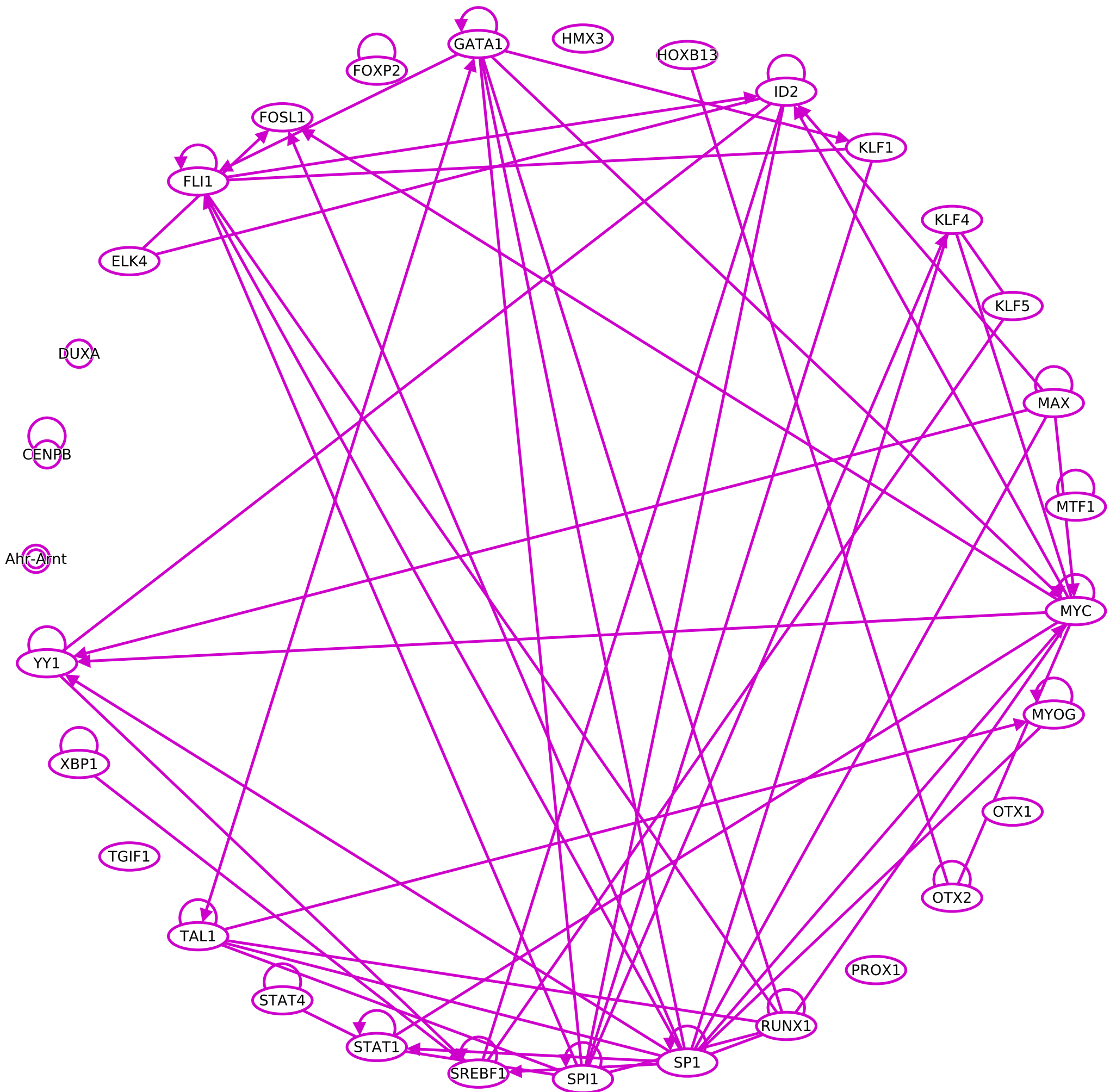

Supplement: Supplementary file 3 — Raw predicted cofactors interaction graphs from Ingenuity Pathway Analysis (IPA). Files with ’_high’ suffix (for high confidence) represent data from “Ingenuity expert findings” and “Experimentally observed” databases. Files with ’_low’ suffix (for low confidence), represent data from all IPA databases. (ZIP 2519 kb) [file 12859_2018_2215_MOESM3_ESM.zip › GATA1_low.pdf]

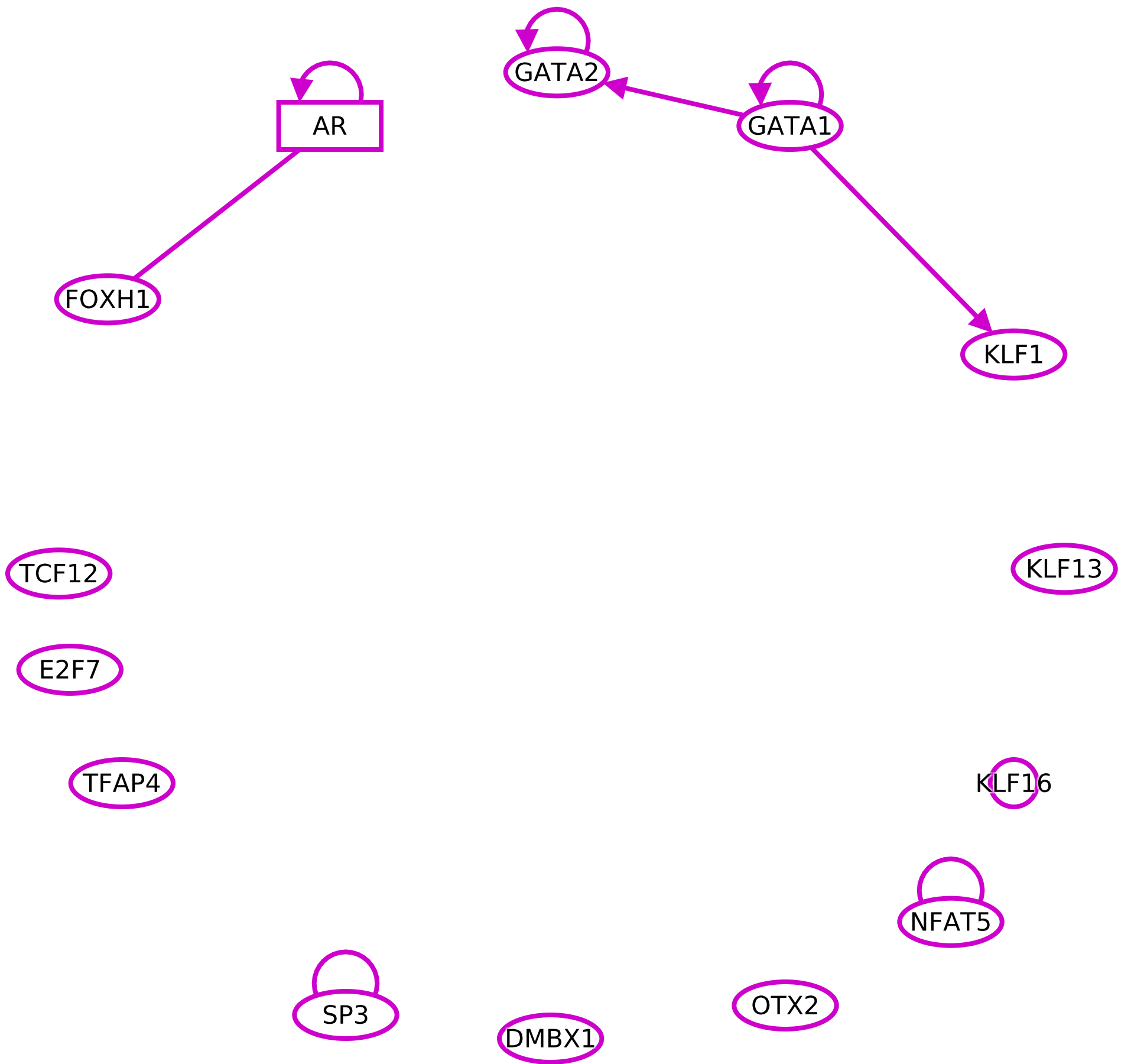

Supplement: Supplementary file 3 — Raw predicted cofactors interaction graphs from Ingenuity Pathway Analysis (IPA). Files with ’_high’ suffix (for high confidence) represent data from “Ingenuity expert findings” and “Experimentally observed” databases. Files with ’_low’ suffix (for low confidence), represent data from all IPA databases. (ZIP 2519 kb) [file 12859_2018_2215_MOESM3_ESM.zip › KLF1_high.pdf]

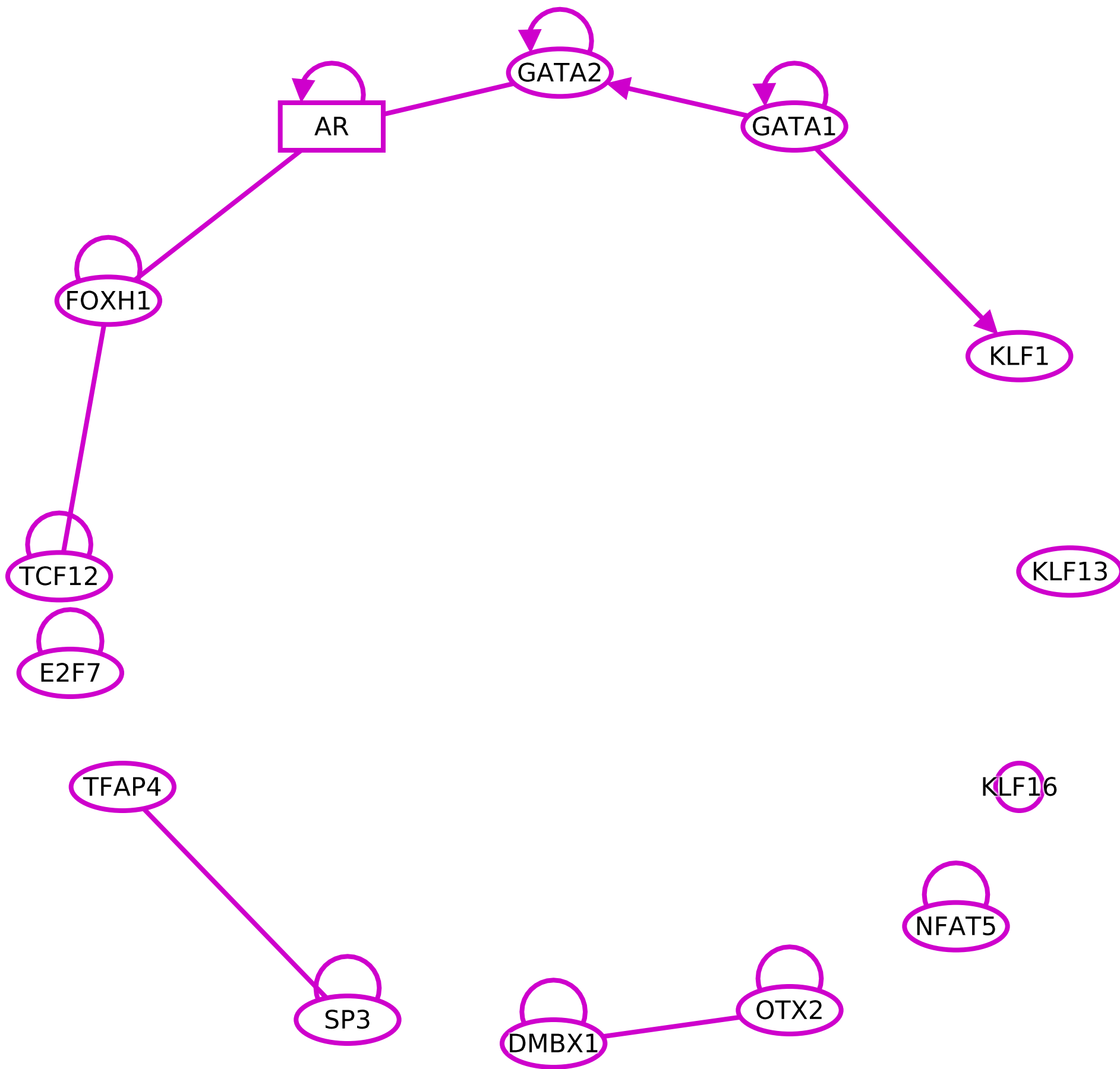

Supplement: Supplementary file 3 — Raw predicted cofactors interaction graphs from Ingenuity Pathway Analysis (IPA). Files with ’_high’ suffix (for high confidence) represent data from “Ingenuity expert findings” and “Experimentally observed” databases. Files with ’_low’ suffix (for low confidence), represent data from all IPA databases. (ZIP 2519 kb) [file 12859_2018_2215_MOESM3_ESM.zip › KLF1_low.pdf]

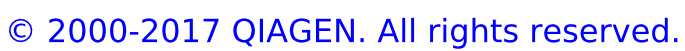

Supplement: Supplementary file 3 — Raw predicted cofactors interaction graphs from Ingenuity Pathway Analysis (IPA). Files with ’_high’ suffix (for high confidence) represent data from “Ingenuity expert findings” and “Experimentally observed” databases. Files with ’_low’ suffix (for low confidence), represent data from all IPA databases. (ZIP 2519 kb) [file 12859_2018_2215_MOESM3_ESM.zip › OCT4_high.pdf]

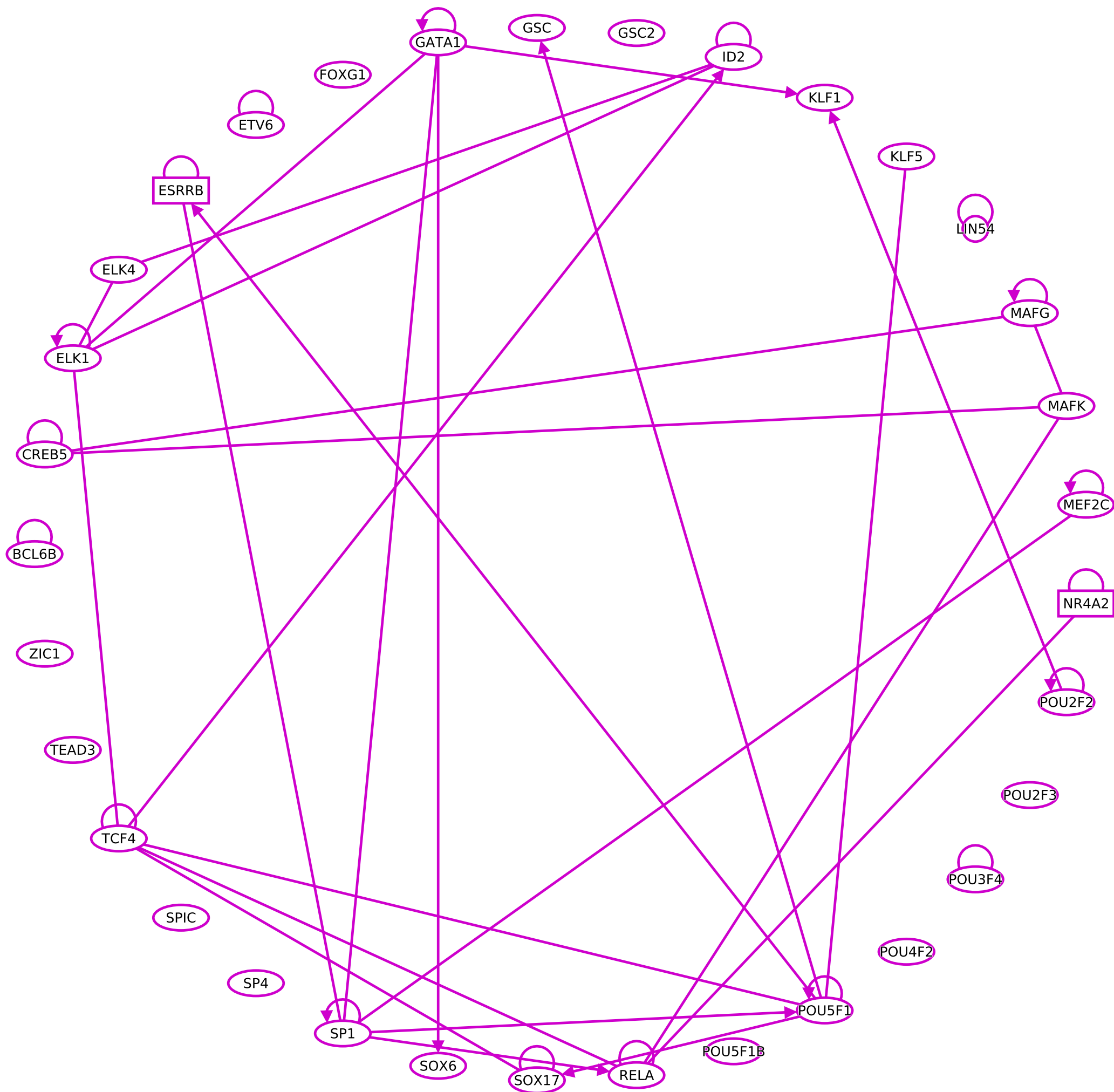

Supplement: Supplementary file 3 — Raw predicted cofactors interaction graphs from Ingenuity Pathway Analysis (IPA). Files with ’_high’ suffix (for high confidence) represent data from “Ingenuity expert findings” and “Experimentally observed” databases. Files with ’_low’ suffix (for low confidence), represent data from all IPA databases. (ZIP 2519 kb) [file 12859_2018_2215_MOESM3_ESM.zip › OCT4_low.pdf]

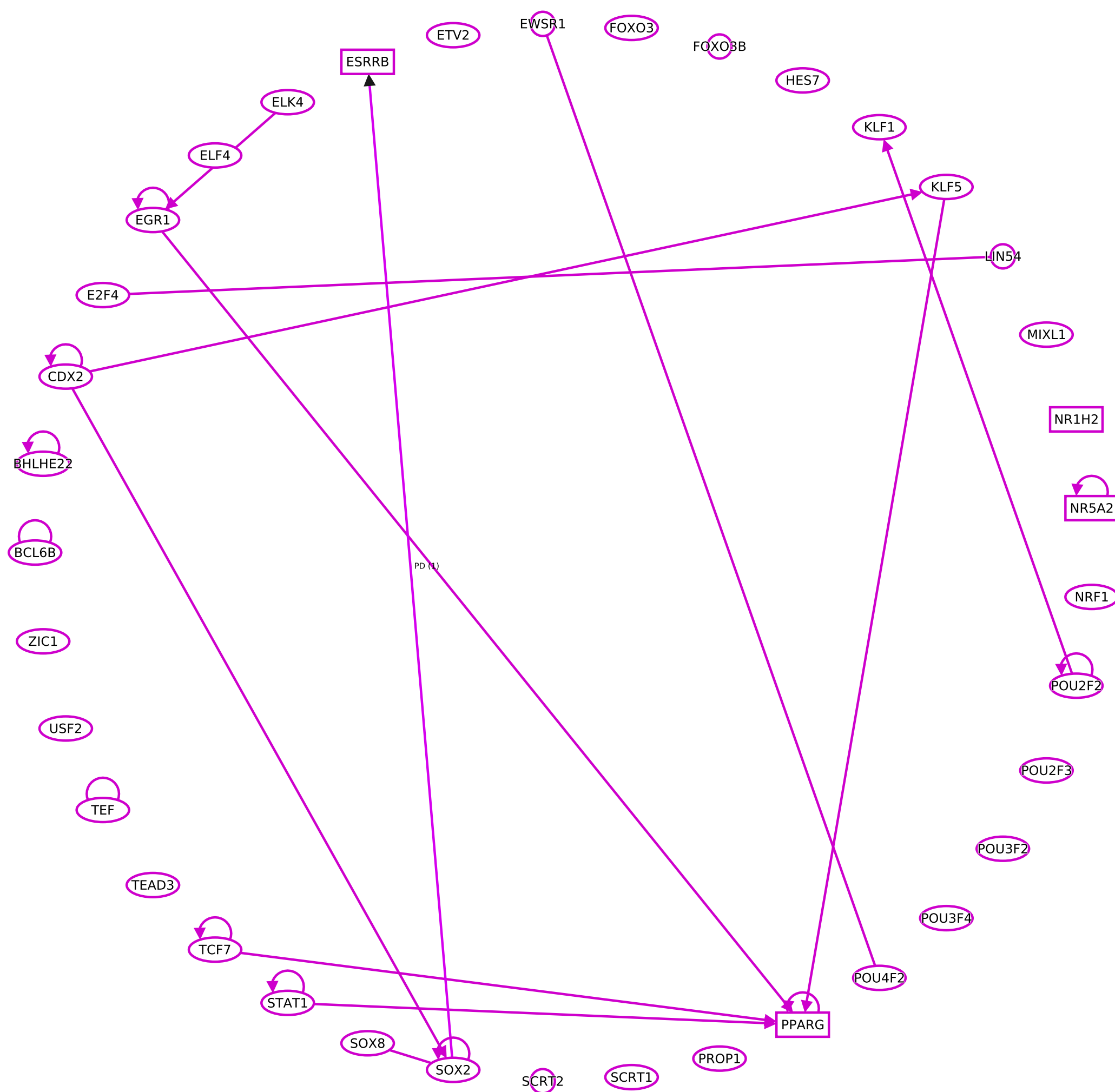

Supplement: Supplementary file 3 — Raw predicted cofactors interaction graphs from Ingenuity Pathway Analysis (IPA). Files with ’_high’ suffix (for high confidence) represent data from “Ingenuity expert findings” and “Experimentally observed” databases. Files with ’_low’ suffix (for low confidence), represent data from all IPA databases. (ZIP 2519 kb) [file 12859_2018_2215_MOESM3_ESM.zip › SOX2_high.pdf]

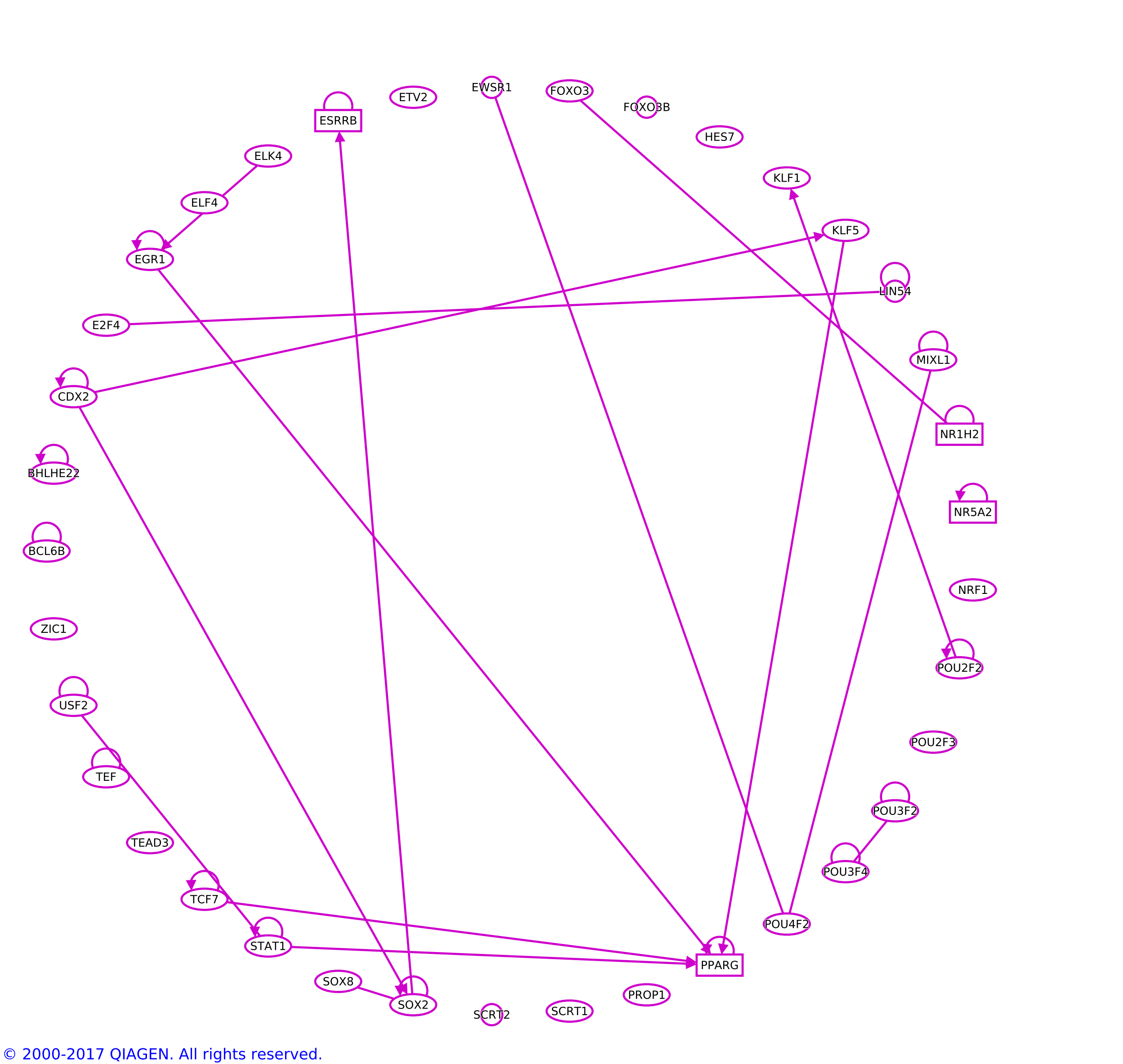

Supplement: Supplementary file 3 — Raw predicted cofactors interaction graphs from Ingenuity Pathway Analysis (IPA). Files with ’_high’ suffix (for high confidence) represent data from “Ingenuity expert findings” and “Experimentally observed” databases. Files with ’_low’ suffix (for low confidence), represent data from all IPA databases. (ZIP 2519 kb) [file 12859_2018_2215_MOESM3_ESM.zip › SOX2_low.pdf]

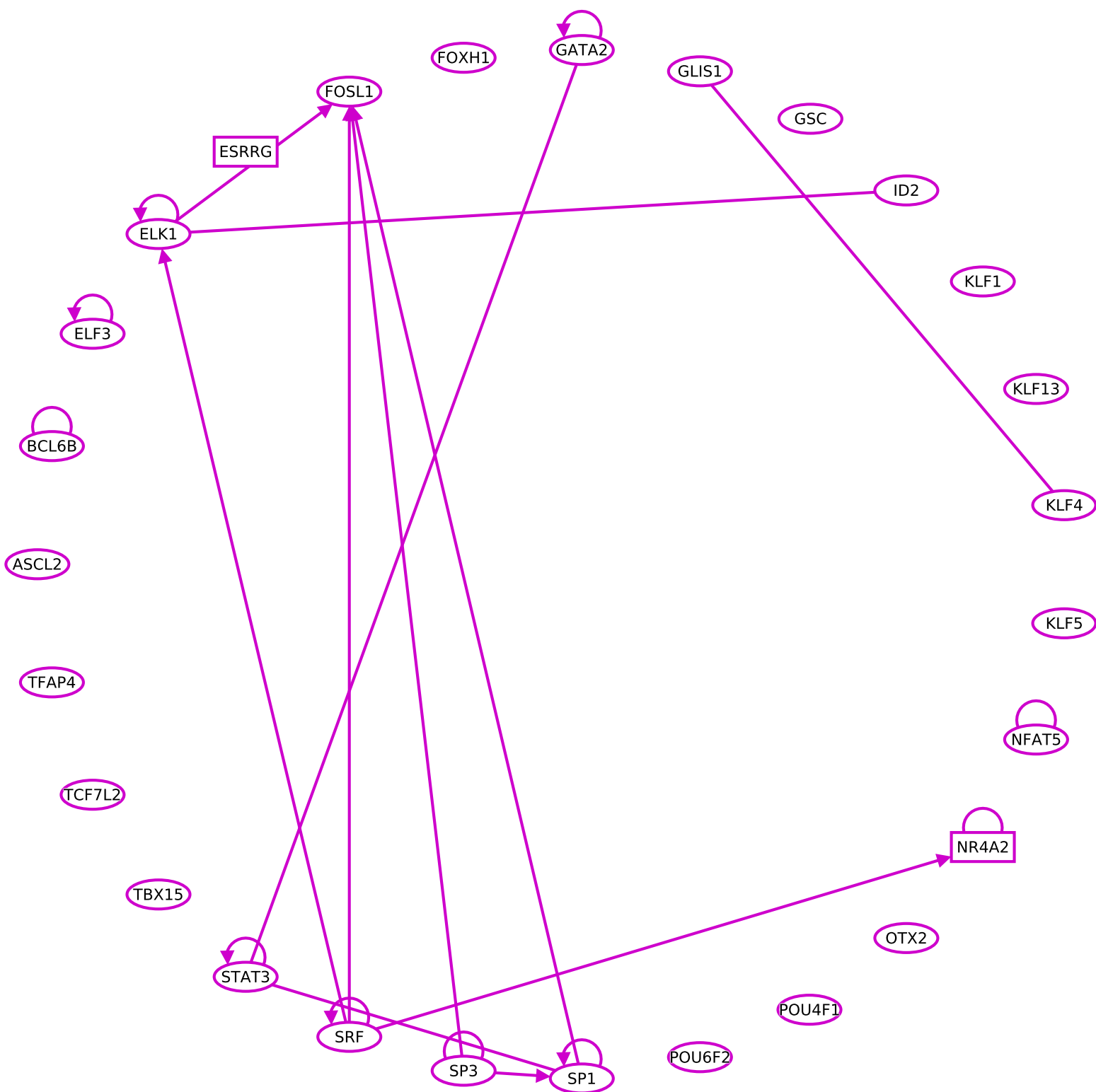

Supplement: Supplementary file 3 — Raw predicted cofactors interaction graphs from Ingenuity Pathway Analysis (IPA). Files with ’_high’ suffix (for high confidence) represent data from “Ingenuity expert findings” and “Experimentally observed” databases. Files with ’_low’ suffix (for low confidence), represent data from all IPA databases. (ZIP 2519 kb) [file 12859_2018_2215_MOESM3_ESM.zip › STAT3_high.pdf]

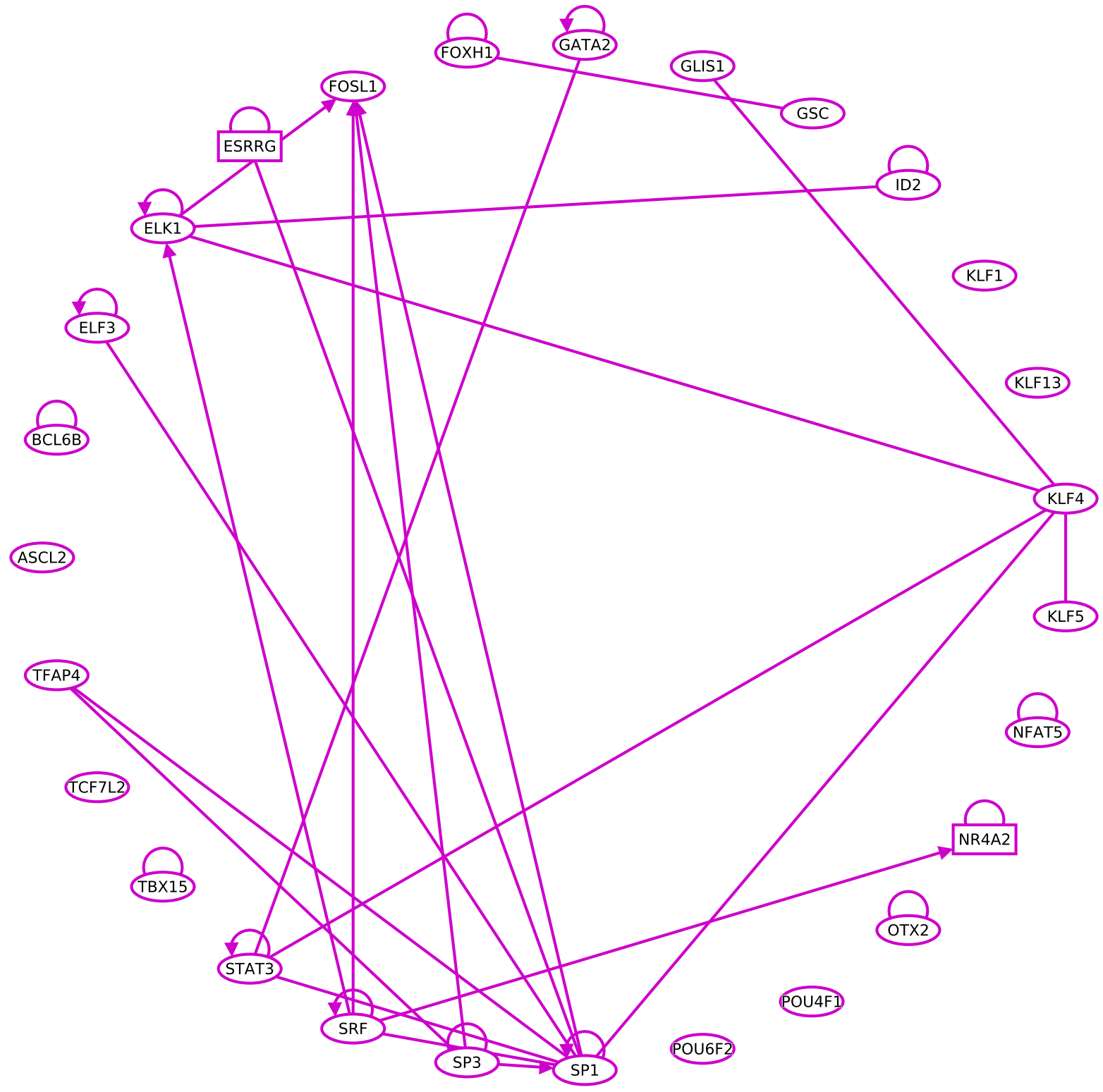

Supplement: Supplementary file 3 — Raw predicted cofactors interaction graphs from Ingenuity Pathway Analysis (IPA). Files with ’_high’ suffix (for high confidence) represent data from “Ingenuity expert findings” and “Experimentally observed” databases. Files with ’_low’ suffix (for low confidence), represent data from all IPA databases. (ZIP 2519 kb) [file 12859_2018_2215_MOESM3_ESM.zip › STAT3_low.pdf]
